# Supplementary material for: Combination Therapy for Neuropathic Pain: A Review of Recent Evidence
Source: J Clin Med. 2021 Aug 11;10(16):3533. doi: 10.3390/jcm10163533 (PMC8396869; doi:10.3390/jcm10163533)
Supplement: Supplementary file 1 [file jcm-10-03533-s001.zip › SUPPLEMENTAL MATERIAL - table s1.pdf]

SUPPLEMENTAL MATERIAL:
Table S1. Secondary outcomes and other evaluations.

	NP symptoms	PGIC	BPI	Rescue medication prescribed / analyzed	concomitant medication	sleep interference	Sensory test	Other	Safety (SAEs) reported (whether it happened or not)	Type of AEs	Drop-out by AEs
Langford, 2013 [43]	?	yes (¬)	yes	yes / no	yes	yes (**)	no	no	yes	yes	yes
Irving, 2012 [44]	no	yes	no	yes / no	yes	no	no	no	yes	yes	yes
Shaibani, 2012 [45]	no	no	no	yes / yes	?	yes	no	no	yes	yes	yes
Tesfaye, 2013 [42]	?	yes	yes	no / no	no	yes	no	no	yes	yes	yes
Holbech, 2015 [38]	yes	no	no	yes / yes	no	no	yes	QST post-hoc	yes	yes	yes
Matsuoka, 2019 [32]	no	no	yes	yes / yes	yes	? (##)	no	no	yes	yes	?
Singh, 2021 [30]	?	no	no	no / no	no	no	#	VDT	no	yes	no
Rigo, 2017 [34]	yes	no	no	yes / ?	yes	yes	no	no	no	yes	yes
Kim, 2016 [35]	no	no	no	no / no	no	no	no	no	no	yes	no
Baron, 2014 [36]	yes	yes	no	? / no	?	yes	no	no	yes	yes	yes
Gilron, 2015 [37]	yes	no	yes	no / no	yes	yes	no	no	no	yes	yes
Pickering, 2020 [31]	yes	yes	yes	no / no	yes	yes	no	no	yes	yes	*
Turcotte, 2015 [39]	no	yes	no	no / no	?	no	no	no	yes	?	yes
Harrison, 2013 [41]	no	no	no	yes / ?	?	no	no	no	yes	yes	yes
Dou, 2017 [33]	no	no	no	yes / ?	no	yes	no	Decrements in morphine	no	yes	yes
Garassino, 2013 [40]	no	no	no	no / no	no	no	no	polymorphism n.s	no	?	yes

This table evaluates other characteristics reported in studies. NP = neuropathic pain, PGIC = patient global impression of change, BPI = brief pain inventory, SAEs = serious adverse events, AEs = adverse events, ¬ = SGIC (subjective global impression of change), ** = measured only by quality, ## = measured by QoL - Quality of Life, * = reported to have zero drop-outs, QST = quantitative sensory testing, VDT = vibration detection threshold, ? = data unclear, n.s = not significant.
