# Supplementary figures and images for: Combination Therapy for Neuropathic Pain: A Review of Recent Evidence
Source: J Clin Med. 2021 Aug 11;10(16):3533. doi: 10.3390/jcm10163533 (PMC8396869; doi:10.3390/jcm10163533)

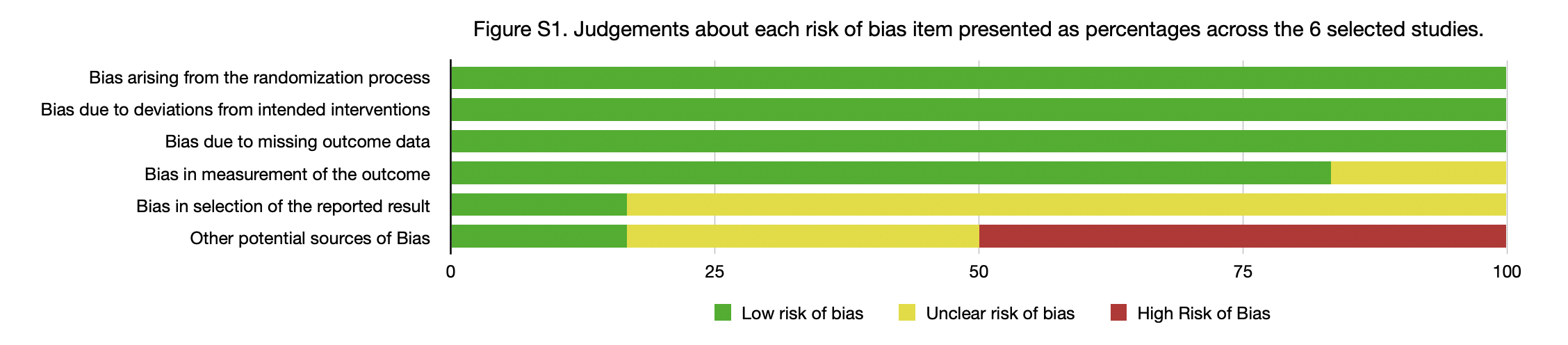

Supplement: Supplementary file 1 [file jcm-10-03533-s001.zip › Figure S1.jpg]

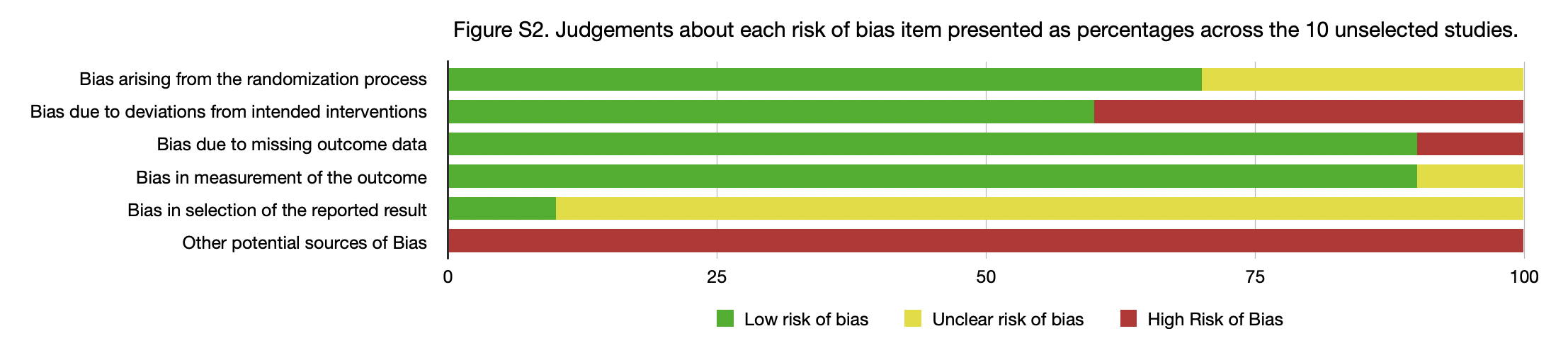

Supplement: Supplementary file 1 [file jcm-10-03533-s001.zip › Figure S2.jpg]
